# Hypersensitivity to amphetamine's psychomotor and reinforcing effects in serotonin transporter knockout rats: Glutamate in the nucleus accumbens

**DOI:** 10.1111/bph.15211

**Published:** 2020-08-30

**Authors:** Lucia Caffino, Michel M. M. Verheij, Karine Roversi, Giorgia Targa, Francesca Mottarlini, Piotr Popik, Agnieska Nikiforuk, Joanna Golebiowska, Fabio Fumagalli, Judith R. Homberg

**Affiliations:** ^1^ Department of Pharmacological and Biomolecular Sciences Università degli Studi di Milano Milan Italy; ^2^ Department of Cognitive Neuroscience, Donders Institute for Brain, Cognition and Behaviour Radboud University Nijmegen Medical Centre Nijmegen The Netherlands; ^3^ Department of Behavioural Neuroscience and Drug Development Maj Institute of Pharmacology, Polish Academy of Sciences Krakow Poland

**Keywords:** amphetamine, glutamate, locomotor activity, nucleus accumbens, self‐administration, serotonin transporter

## Abstract

**Background and Purpose:**

Amphetamine (AMPH) use disorder is a serious health concern, but, surprisingly, little is known about the vulnerability to the moderate and compulsive use of this psychostimulant and its underlying mechanisms. Previous research showed that inherited serotonin transporter (SERT) down‐regulation increases the motor response to cocaine, as well as moderate (as measured during daily 1‐h self‐administration sessions) and compulsive (as measured during daily 6‐h self‐administration sessions) intake of this psychostimulant. Here, we sought to investigate whether these findings generalize to AMPH and the underlying mechanisms in the nucleus accumbens.

**Experimental Approach:**

In serotonin transporter knockout (SERT^−/−^) and wild‐type control (SERT^+/+^) rats, we assessed the locomotor response to acute AMPH and i.v. AMPH self‐administration under short access (ShA: 1‐h daily sessions) and long access (LgA: 6‐h daily sessions) conditions. Twenty‐four hours after AMPH self‐administration, we analysed the expression of glutamate system components in the nucleus accumbens shell and core.

**Key Results:**

We found that SERT^−/−^ animals displayed an increased AMPH‐induced locomotor response and increased AMPH self‐administration under LgA but not ShA conditions. Further, we observed changes in the vesicular and glial glutamate transporters, NMDA and AMPA receptor subunits, and their respective postsynaptic scaffolding proteins as function of SERT genotype and AMPH exposure (baseline, ShA, and LgA), specifically in the nucleus accumbens shell.

**Conclusion and Implications:**

We demonstrate that SERT gene deletion increases the psychomotor and reinforcing effects of AMPH and that the latter is potentially mediated, at least in part, by homeostatic changes in the glutamatergic synapse of the nucleus accumbens shell and/or core.

## Graphical abstract

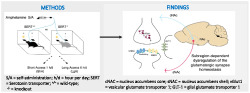

**Abbreviations**
5‐HTTLPR: serotonin transporter‐linked polymorphic region
AMPH: amphetamine
cNAc: nucleus accumbens core
FR1: fixed ratio 1
GG: Greenhouse–Geisser
GLT‐1: glial glutamate transporter 1
GluA1/A2: glutamate AMPA receptor 1/2 subunit
GluN1/2A/2B: glutamate NMDA receptor 1/2A/2B subunit
GRIP: glutamate receptor interacting protein; AMPA receptor scaffolding protein
LgA: long access (6‐h daily self‐administration sessions)
NAc: nucleus accumbens
PFC: prefrontal cortex
SAP102: synapse associated protein 102; NMDA receptor scaffolding protein
SAP97: synapse associated protein 97; AMPA receptor scaffolding protein
SERT^−/−^: serotonin transporter knockout
SERT^+/+^: wild‐type counterpart of serotonin transporter knockout rat
ShA: short access (1‐h daily self‐administration sessions)
sNAc: nucleus accumbens shell
vGlut1: vesicular glutamate transporter 1

**What is already known**

- Rats lacking the serotonin transporter show increased moderate and compulsive cocaine self‐administration.

**What this study adds**

- Serotonin transporter knockout rats show also increased compulsive, but not moderate, amphetamine self‐administration.
- This is associated with glutamatergic synaptic changes in the nucleus accumbens shell and core.

**What is the clinical significance**

- We provide a mechanism for amphetamine addiction proneness in individuals with inherited serotonin transporter down‐regulation.

## 1 INTRODUCTION

Amphetamine (AMPH) is a psychostimulant substance widely used across the world because of its euphoric effects, making it a remaining public health issue (United Nations Office on Drugs and Crime, 2018). One in 10 AMPH users develops dependency on the drug (Anthony, Warner, & Kessler, 1994). However, up to date, it is not clear which factors shape vulnerability to AMPH dependency, and the underlying mechanisms are still elusive. This would be critical as such insight ultimately would help to inform early interventions or treatments.

In humans, the short (s) low expression allelic variant of the serotonin transporter‐linked polymorphic region (5‐HTTLPR) has been considered as risk allele for AMPH and cocaine addiction (Cao, Hudziak, & Li, 2013). Accordingly, we and others previously observed that serotonin transporter knockout (SERT ^−/−^) rats, which may model the 5‐HTTLPR s‐allele (Holmes, Bogdan, & Pizzagalli, 2010; Schipper et al., 2019) in humans, are more sensitive to the psychomotor and reinforcing effects of these psychostimulants (Homberg et al., 2008; Oakly, Brox, Schenk, & Ellenbroek, 2014; Pettie, Oakly, Harper, & Ellenbroek, 2019; Verheij et al., 2018). However, as to whether these observations generalize to AMPH is currently not known.

Mechanistically, psychostimulants increase extracellular levels of dopamine, serotonin, and noradrenaline via an action on their respective transporter, although with varying sensitivity for the different psychostimulants. In particular, cocaine and AMPH are thought to mediate their reward‐related effects by increasing dopamine levels (Koob, 1992; Lile & Nader, 2003; Natarajan & Yamamoto, 2011; Rocha et al., 1998; Rothman & Glowa, 1995; Woolverton & Johnson, 1992). Yet we observed that cocaine more strongly increased locomotor activity in SERT^−/−^ versus their wild‐type counterparts (SERT^+/+^) (Homberg et al., 2008). In addition, on top of high baseline levels of extracellular serotonin (Homberg et al., 2007), the cocaine‐induced increase of extracellular serotonin, but not of extracellular dopamine and noradrenaline, was reduced in the nucleus accumbens (NAc) of SERT^−/−^rats (Verheij, Karel, Cools, & Homberg, 2014). This suggests that SERT and accumbal serotonin may play a critical role in the psychomotor and reinforcing effects of psychostimulants. The role of SERT, however, may differ between psychostimulants, since AMPH has a lower sensitivity for SERT than cocaine (Howell & Kimmel, 2008). Given the previously observed SERT‐dependent changes in the psychomotor and reinforcing effects of cocaine, we have now analysed the SERT‐dependent psychomotor and reinforcing effects of AMPH. To this end, the acute locomotor response to various doses of AMPH, as well as AMPH self‐administration under short access (ShA; 1 h·day^−1^) conditions (modelling moderate drug use) and long access (LgA; 6 h·day^−1^) conditions (modelling uncontrolled compulsive drug use) (Ahmed & Koob, 1998) has been obtained in SERT^−/−^ and their wild‐type control rats.

From a neurobiological point of view, the transition from goal‐directed drug intake towards addictive states has been proposed to result from a shift of control of motivated behaviour from the prefrontal cortex (PFC) to the striatum via glutamatergic projections (Everitt & Robbins, 2016; Kalivas & Volkow, 2005). We have recently shown that the interaction between serotonin and cocaine intake, as well as the transition from hedonic to compulsive use of the psychostimulant, dysregulates glutamatergic synapses in the habenula and PFC (Caffino et al., 2019, 2020), pointing to serotonin–glutamate interactions as a neurobiological substrate of heightened vulnerability to drug dependence. Yet, surprisingly, little is known about the AMPH‐induced neuroadaptations on glutamate signalling and the serotoninergic control on glutamatergic homeostasis in the transition from hedonic to compulsive AMPH use (Faraone, 2018). Interestingly, it has been demonstrated that AMPH reduced striatal protein expression of metabotropic mGluR5 receptor in rats (Shaffer, Guo, Fibuch, Mao, & Wang, 2010), suggesting that altered glutamate signalling can contribute to altered sensitivity to the psychomotor and reinforcing effects of AMPH.

The NAc is a brain region critical for the neuroadaptive changes subserving drug intake, withdrawal, and motivation to search for drugs. This brain region is characterized by the above‐mentioned SERT‐dependent changes in the serotonin response to psychostimulants and a strong glutamatergic signalling. On this basis, we sought to determine the SERT‐dependent effects of AMPH self‐administration on the accumbal glutamatergic synapse. To this end, we deeply analysed the glutamate synapse in the NAc. Specifically, because the nucleus accumbens core subregion (cNAc) is selectively involved in incubation of drug seeking, whereas the nucleus accumbens shell (sNAc) is involved in escalation of drug taking (Guillem, Ahmed, & Peoples, 2014), we investigated glutamate homeostasis in both subregions and focused on (a) the mechanisms regulating the storage of glutamate in presynaptic vesicles, which is mediated by the vesicular glutamate transporter 1 (vGlut1) (El Mestikawy, Wallen‐Mackenzie, Fortin, Descarries, & Trudeau, 2011), (b) the clearance of the neurotransmitter from the synaptic cleft by the glutamate transporter 1 (GLT‐1) (Roberts‐Wolfe & Kalivas, 2015), as well as (c) the main glutamate postsynaptic receptors (NMDA and AMPA) (Traynelis et al., 2010) and (d) the main scaffolding proteins of NMDA (SAP102) and AMPA (SAP97 and GRIP) glutamate receptors, whose job is to firmly anchor these receptors to the membrane (Oliva, Escobedo, Astorga, Molina, & Sierralta, 2012).

## 2 METHODS

Animal studies are reported in compliance with the ARRIVE guidelines (Kilkenny, Browne, Cuthill, Emerson, & Altman, 2010; McGrath & Lilley, 2015) and with the recommendations made by the *British Journal of Pharmacology.*

### 2.1 Animals

We employed rats in this study because the rat is the preferred species for preclinical addiction research (Homberg, Wohr, & Alenina, 2017). We used SERT^−/−^ rats as model for the human SERT gene polymorphism as has been established previously (Caspi, Hariri, Holmes, Uher, & Moffitt, 2010). SERT^−/−^ rats (SLC6A4^1Hubr^) were generated by *N*‐ethyl‐*N*‐nitrosourea‐induced mutagenesis (Homberg et al., 2007; Smits et al., 2006) and outcrossed with commercially available Wistar rats (Harlan, Ter Horst, the Netherlands) for at least 15 generations. Male SERT^−/−^ and SERT^+/+^ offspring were used for experiments as in human psychostimulant addiction has a clear male preponderance (Degenhardt et al., 2014). The rats were bred in Nijmegen and either tested in Nijmegen or transported to Poland. In Nijmegen, rats were housed in groups of two rats in enriched Macrolon type III cages (42 × 26 × 15 cm; Techniplast 1291H, Tecnilab‐BMI) with corncobs bedding (irradiated, SPPS COB12, Bio Services) under conventional conditions (no filtertops). The animals had access to food (dried pellets of standard chow food [Ssniff RM V1534‐703 diet supplied by Bio Services]) and water ad libitum, except during test phases. The rats were housed under a reversed day and night cycle (lights off at 08:00 a.m.) in temperature‐ (21 ± 1°C) and humidity‐controlled (55% ± 5%) rooms. Testing (AMPH self‐administration) took place in the dark phase of the light/dark cycle. In Poland, rats were group‐housed four rats per cage (380 × 200 × 590 mm; Ehret Labor‐ und Pharmatechnik GMBH & Co.KG, Germany) with aspen litter (MIDI LTE E‐002 Abedd, AnimaLab, Poland) and environmental enrichment. The rats had free access to food (VRF1 [P] Special Diets Services, England) and water. Animals were housed in a temperature‐controlled (21 ± 1°C) and humidity‐controlled (40%–50%) colony room under a 12/12 h light/dark cycle (lights on at 06:00 a.m.). Behavioural testing (locomotor activity test) was performed during the light phase of the light/dark cycle. Testing was always done blindly by an experimenter who was unaware of the genotype of the animals. Animals were not randomized, as their genotype determined the group in which the animals were placed. All procedures were carried out in agreement with the current Directive 2010/63/EU and Dutch and Polish Research Council Guide for the Care and Use of Laboratory Animals and were approved by local and national Institutional Animal Care and Use Committees. The experimental procedures at Radboudumc (Nijmegen, the Netherlands) were performed under a project licence from the Central Committee on Animal Experiments (Centrale Commissie Dierproeven, The Hague, the Netherlands), in full compliance with the legal requirements of Dutch legislation on the use and protection of laboratory animals (Animal Testing Act). The experimental procedures in Poland were performed at the Maj Institute of Pharmacology, Polish Academy of Science, Krakow, Poland. All efforts were made to reduce the number of animals used and their suffering both in the Netherlands and in Poland.

### 2.2 Drugs

Dex(d)‐amphetamine was provided by Spruyt Hillen (IJsselstein, the Netherlands) and was dissolved in saline 0.9%.

### 2.3 Behaviour

#### 2.3.1 AMPH‐induced locomotor activity

**FIGURE 1:**
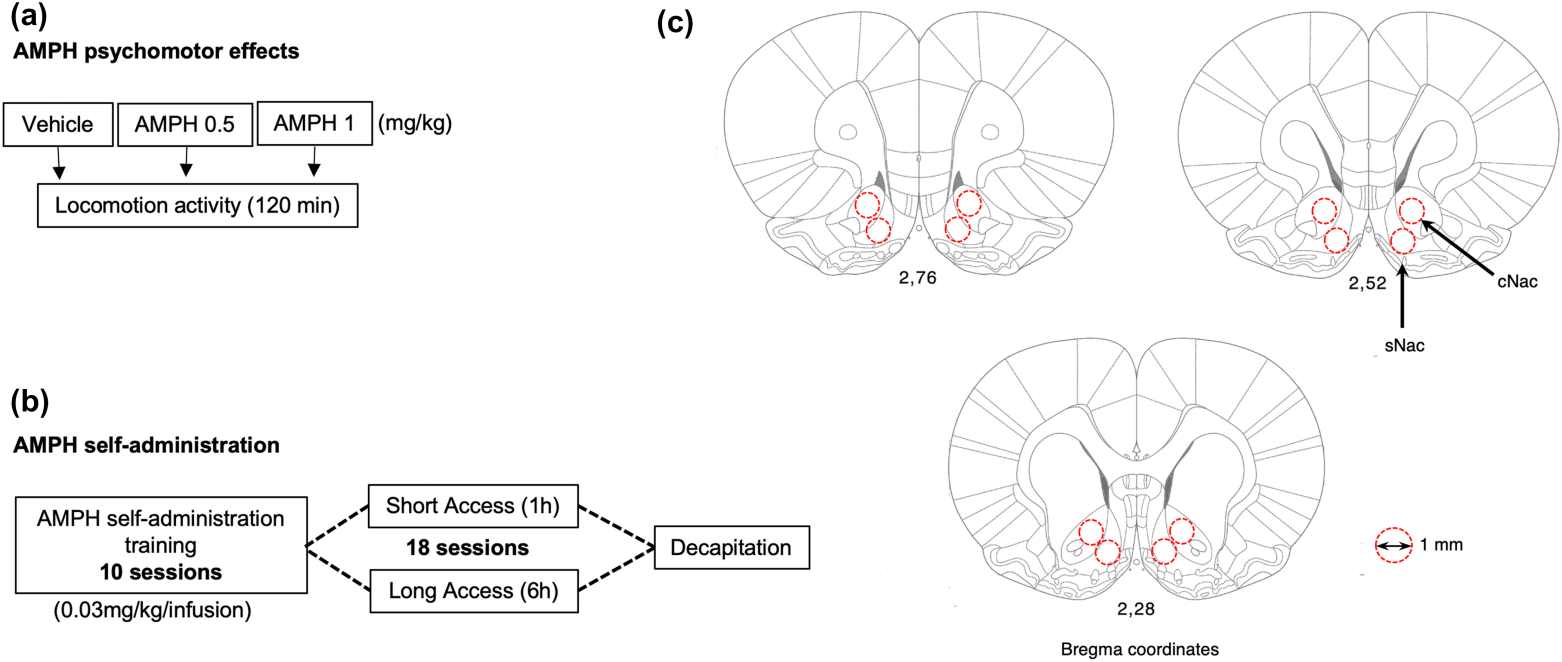
Schematic representation of the experimental paradigm performed in SERT^−/−^ and SERT^+/+^ rats. (a) The psychomotor effects of AMPH were assessed immediately after AMPH injection and evaluated for 120 min. (b) AMPH self‐administration training was performed during 10 days, after which the animals were subdivided into short‐ (ShA) or long‐access (LgA) groups. During the next 18 days the rats were allowed to self‐administer AMPH under either of these conditions. (c) Twenty‐four hours after the final ShA or LgA session, rats were decapitated, and punches from the nucleus accumbens core (cNAc) and shell (sNAc) subregions (localized using the Paxinos & Watson, 2007 edition of the rat brain atlas) were collected for gene expression analyses of the glutamatergic synapse

The psychomotor effects of AMPH were tested in a grey plywood open field of 50 × 50 × 50 cm, which was illuminated by ~20 Lux. After 30 mins of habituation, rats, weighing between 500 and 600 g (age: 6–7 months), were treated with 0.5 mg·kg^−1^ AMPH (SERT^+/+^: *n* = 11; SERT^−/−^: *n* = 12), 1 mg·kg^−1^ AMPH (SERT^+/+^: *n* = 13; SERT^−/−^: *n* = 13), or vehicle (SERT^+/+^: *n* = 24; vehicle SERT^−/−^: *n* = 25) according to a standard Latin square design using the same animals with a break of 4 weeks between the various treatments. Distance moved was recorded for 120 mins (Figure 1a) and analysed afterwards using the ANY‐maze video tracking system (version 4.82) from Stoelting.

### 2.4 AMPH self‐administration

#### 2.4.1 Intravenous catheterization

Under isoflurane aesthesia, SERT^+/+^ and SERT^−/−^ rats, weighing between 300 and 450 g (age: 3–5 months), received a micro‐Renathane catheter into the right external jugular vein (for details, see: Wee, Specio, & Koob, 2007). The tubing (0.037″ o.d. × 0.023″ i.d.; Braintree Scientific Inc., USA) was guided s.c. to a stainless steel cannula (22G, Plastics One Inc., USA) at the back of the rat. After surgery, rats were given Flunixin (analgesic drug: 2.5 mg·ml^−1^·kg^−1^, s.c.) and Cefazolin (antibiotic drug: 15 mg·ml^−1^·kg^−1^) daily for respectively 3 and 7 days. After surgery, rats were singly housed to prevent damage to the cannula due to cage mates. These rats received a wooden block and nest material as cage enrichment but not a shelter to prevent collisions with the cannula. The body weight of the animals was monitored daily, and full recovery of body weight happened within 3 to 5 days. During recovery, no unexpected changes in animal behaviour (e.g., no self‐grooming and no water intake) were observed. Catheter patency was maintained by daily flushing with 0.2 ml heparinized saline. In case of obstruction of the catheter, the animal was removed from the study.

### 2.5 Self‐administration chambers

After, at least, 1 week of recovery, AMPH self‐administration was performed in standard operant boxes (28 × 26 × 20 cm; Med Associates Inc., USA). The cannula was connected to a swivel system that allowed the animal to move freely. Once the animals were connected to the system, two retractable levers were presented. Pressing the active (correct) lever resulted in i.v. infusion of AMPH (volume: 0.1 ml in 3 s). Furthermore, a light cue above the active lever indicated a timeout period of 20 s during which no AMPH infusion was available. Pressing the inactive (incorrect) lever had no programmed consequences.

### 2.6 Intravenous AMPH self‐administration training

Rats were exposed to 1‐h daily training sessions (0.03 mg·kg^−1^ per infusion) under a fixed ratio 1 (FR1) schedule over the course of 10 days. During training, the maximum number of infusions was set at 20 to prevent an early overdose.

### 2.7 ShA and LgA AMPH self‐administration

After training, rats were divided into two groups with an equal number of AMPH injections during the last three training session. One group continued to self‐administer AMPH (0.03 mg·kg^−1^ per infusion) for 1 h·day^−1^ (the ShA group: SERT^+/+^ *n* = 14; SERT^−/−^ *n* = 13), and the other group self‐administered the same dose for 6 h·day^−1^ (the LgA group: SERT^+/+^ *n* = 14; SERT^−/−^ *n* = 13) (see also Gipson & Bardo, 2009). Protein expression data of these groups were compared to a new group of animals that was subjected to surgery but were not exposed to AMPH (the AMPH‐naive group: SERT^+/+^ *n* = 6; SERT^−/−^ *n* = 6; Figure 1b). The animals were tested daily between 8 a.m. and 6 p.m. for 18 consecutive days (see also Verheij et al., 2018), and the order of testing ShA and LgA groups was changed every day. Due to a limited number of self‐administration chambers, self‐administration was performed in two cohorts of animals (age‐matched rats of both genotypes were always tested simultaneously and in equal numbers). The intake did not differ between batches of animals.

### 2.8 Collection of NAc tissue

Twenty‐four hours after the last ShA or LgA session, rats were decapitated without anaesthesia. This procedure was used to avoid effects of anaesthetics on gene expression and is in accordance of the Dutch legal regulations for killing rodents. Brains were collected, freshly frozen, and stored at −80°C. cNAc and sNAc punches were collected from 220‐μm brain slices, as previously described (Giannotti, Caffino, Mottarlini, Racagni, & Fumagalli, 2016). The punches were collected according to coordinates as described by Paxinos and Watson (2007) (from bregma +2.76 to bregma +0.84 mm), using a punching needle with a diameter of 1 mm (Figure 1c). For both regions, the two hemispheres were pooled.

### 2.9 Protein extraction and western blot analyses

The immuno‐related procedures used comply with the recommendations made by the *British Journal of Pharmacology* (Alexander et al., 2018).

Bilateral punches of cNAc and sNAc of 63 animals were sonicated using a cold buffer containing 0.32‐M sucrose, 1‐mM HEPES solution, 0.1‐mM EGTA, and 0.1‐mM PMSF, pH = 7.4, in the presence of a complete set of protease inhibitors and a phosphatase inhibitor cocktail. Total proteins have been measured in the whole homogenate by the Bio‐Rad Protein Assay (Bio‐Rad Laboratories). Western blots were run as previously described (Caffino, Giannotti, Mottarlini, Racagni, & Fumagalli, 2017). Briefly, 10 μg of proteins for each sample was run on an 8% SDS‐PAGE under reducing conditions and then electrophoretically transferred onto nitrocellulose membranes (GE Healthcare, Milan, Italy). Blots were blocked 1 h at room temperature with I‐Block solution (Life Technologies Italia, Italy) in tris‐buffered saline + 0.1% Tween‐20 buffer and then incubated with antibodies against the total proteins of interest.

The conditions of the primary antibodies were the following:

anti‐vGlut1 (1:1000; Cell Signaling Technology Inc.; RRID:AB_2797887), anti‐GLT1 (1:5000; AbCam; RRID:AB_1566262), anti‐GluN1 (1:1000; Invitrogen; RRID:AB_2533060), anti‐GluN2B (1:1000; Santa Cruz Biotechonology; RRID:AB_670229), anti‐GluN2A (1:1000; Invitrogen; RRID:AB_2536209), anti‐SAP102 (1:1000; Cell Signaling Technology Inc.; RRID:AB_2799325), anti‐GluA1 (1:2000; Cell Signaling Technology Inc.;, RRID:AB_2732897), anti‐GluA2 (1:2000; Cell Signaling Technology Inc.; RRID:AB_10622024), anti‐SAP97 (1:1000; AbCam; RRID:AB_2091910), anti‐GRIP (1:2000; Synaptic System; RRID:AB_887728), and anti‐β‐Actin (1:10000; Sigma‐Aldrich; RRID:AB_476697).

Expression levels of every single protein was normalized using its own β‐actin loading control, which was detected by evaluating the band density at 43 kDa. Immunocomplexes were visualized by chemiluminescence using the Chemidoc MP Imaging System (Bio‐Rad Laboratories; RRID:SCCR_014210). Due to the high number of samples, they were divided across three gels that were run simultaneously and analysed. Aliquots of samples collected from SERT^+/+^‐naïve rats were distributed across the various gels (A–C) and used as reference values. We used the following correction factor to the different gels: correction factor gel B = average of (OD protein of interest/OD β‐actin for each sample loaded in gel A)/(OD protein of interest/OD β‐actin for the same sample loaded in gel B). Correction factor gel C = average of (OD protein of interest/OD β‐actin for each sample loaded in gel A)/(OD protein of interest/OD β‐actin for the same sample loaded in gel C). By calculating this correction factor, we were able to evaluate genotype and AMPH exposure as independent variables despite the high number of samples. Gels were run two times each, and the results represent the average from two different runs.

### 2.10 Data and statistical analysis

The data and statistical analysis comply with the recommendations of the *British Journal of Pharmacology* on experimental design and analysis in pharmacology (Curtis et al., 2018). Previous self‐administration studies in cocaine‐treated SERT^−/−^ and SERT^+/+^ animals have revealed a significant genotype × self‐administration session effect with an effect size of about 0.4 ηp^2^ (see also Verheij et al., 2018). Entering this effect size (based on the previously obtained SD), together with an alpha value of 0.05 and a power of 0.80, into G*power (version 3.1), revealed a minimum number of 13–14 rats per genotype. Because of the substantially smaller SD typically observed in our molecular studies (e.g., Caffino et al., 2019, 2020), the number of animals in the AMPH‐naive groups that were not exposed to self‐administration were reduced to 6. All animals tested were treated as independent values; there were no technical replicates.

#### 2.10.1 Behavioural data

The Kolmogorov–Smirnov test was employed to determine normality of residuals, and no significant variance in homogeneity was found. The psychomotor data were checked for outliers using the Grubbs method (data points with *P* < 0.05 were considered outliers). Two outliers were removed from the analysis (1 SERT^+/+^ rat and 1 SERT^−/−^ rat). Because of their longitudinal nature, over 28 daily sessions, the AMPH self‐administration data were not subjected to an outlier test. To analyse the AMPH‐induced psychomotor responses, a three‐way ANOVA with the factors genotype, AMPH dose, and time (for repeated measures) was used. To analyse the AMPH self‐administration data, a number of rewards were analysed using a three‐way ANOVA with the factor genotype, access type, and self‐administration session (for repeated measures). Sphericity of the variance matrix was checked using the Mauchly test. Data analysis was Greenhouse–Geisser (GG) corrected when sphericity was violated. Statistics were performed in IBM SPSS statistics version 23. Student's *t*‐test was conducted as post hoc test when allowed. Significance for all tests was assumed at *P* < 0.05. *P* values are given in the figure legends. Complete statistics can be found in Table S1.

#### 2.10.2 Molecular data

The Kolmogorov–Smirnov test was employed to determine normality of residuals, and no significant variance in homogeneity was found. Thus, the molecular changes produced by genotype and AMPH exposure alone as well as by their combination were analysed using a two‐way ANOVA, with the factors genotype and type of AMPH access as independent variables. When dictated by significant (*P* < 0.05) interaction terms, Tukey's multiple comparisons test was used to characterize differences among individual groups of rats. Two‐way ANOVA analyses were performed using absolute data. Then, data were normalized as percentages of the AMPH‐naïve SERT^+/+^ control rats that were not exposed to either AMPH ShA or LgA to enable visual comparisons across genotypes with different degrees of expression of glutamatergic molecular determinants. Values are presented as percentage of control rats. Subjects were eliminated from the final data set if their data deviated from the mean by 2 SDs. Prism 6.0 (GraphPad; RRID:SCR_002798) was used to analyse all the data. Data are shown as mean ± SEM and as % of baseline to control for unwanted sources of variation. Significance for all tests was assumed at *P* < 0.05. *P* values are mentioned in the figure legends. Complete statistics can be found in Table S1.

### 2.11 Nomenclature of targets and ligands

Key protein targets and ligands in this article are hyperlinked to corresponding entries in http://www.guidetopharmacology.org, the common portal for data from the IUPHAR/BPS Guide to PHARMACOLOGY (Harding et al., 2018), and are permanently archived in the Concise Guide to PHARMACOLOGY 2019/20 (Alexander et al., 2019).

## 3 RESULTS

### 3.1 The psychomotor effects of AMPH in SERT^+/+^ and SERT^−/−^ rats

**FIGURE 2:**
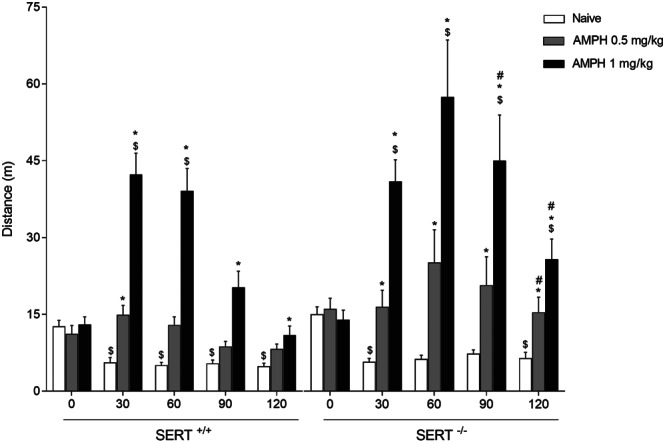
Psychomotor effects of AMPH in SERT^+/+^ and SERT^−/−^ animals. The AMPH‐induced and dose‐dependent increase in locomotor activity was stronger in SERT^−/−^ versus SERT^+/+^ rats. Data represent mean ± SEM distance moved. ^*^ *P* < 0.05 versus respective vehicle; ^#^ *P* < 0.05 in AMPH SERT^−/−^ rats versus AMPH SERT^+/+^ rats; ^$^ *P* < 0.05 versus respective baseline (time 0)

AMPH resulted in a dose‐dependent increase in locomotor activity over time (Figure 2: AMPH dose × time effect). This increase in locomotor activity was stronger in SERT^−/−^ compared to SERT^+/+^ rats (Figure 2: genotype × AMPH dose × time effect).

### 3.2 AMPH self‐administration in SERT^−/−^ and SERT^+/+^ rats

**FIGURE 3:**
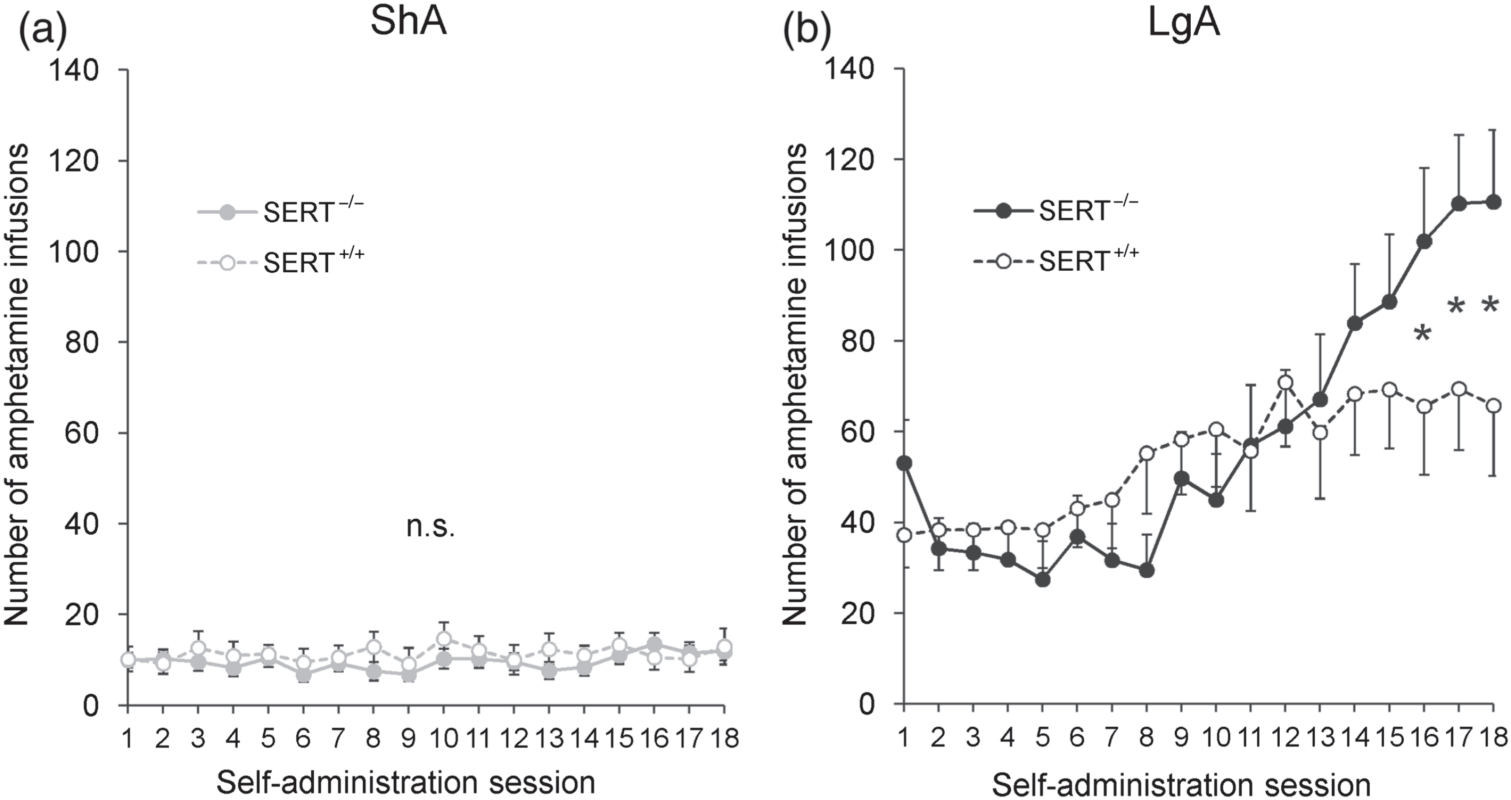
AMPH self‐administration in SERT^+/+^ and SERT^−/−^ animals. The escalation of AMPH intake over the self‐administration sessions was larger in rats under LgA than under ShA conditions (a versus b). The daily AMPH intake was larger in SERT^−/−^ versus SERT^+/+^ rats under (b) LgA, but not (a) ShA conditions. Data are represented as mean ± SEM. n.s., not significant. ^*^ *P* ≤ 0.05 (one‐sided *t*‐test) versus SERT^+/+^

As expected, the escalation of AMPH intake over the daily self‐administration sessions was higher in LgA than ShA rats (Figure 3a vs. Figure 3b: access × self‐administration session effect) and influenced by SERT genotype (Figure 3a,b: genotype × access × self‐administration session effect). More specifically, SERT^−/−^ rats were found to increase the daily intake of AMPH under LgA (Figure 3b: genotype × self‐administration session effect) but not under ShA conditions (Figure 3a: genotype × self‐administration session effect). No differences between SERT^−/−^ and SERT^+/+^ rats were found for the number of incorrect lever presses (Figure S1a,b: genotype (× self‐administration session) effect: not significant) and timeout responses (Figure S1c,d: genotype (× self‐administration session) effect: not significant).

### 3.3 Effect of ShA and LgA AMPH self‐administration on the glutamate presynaptic terminal in SERT^+/+^ and SERT^−/−^ rats

**FIGURE 4:**
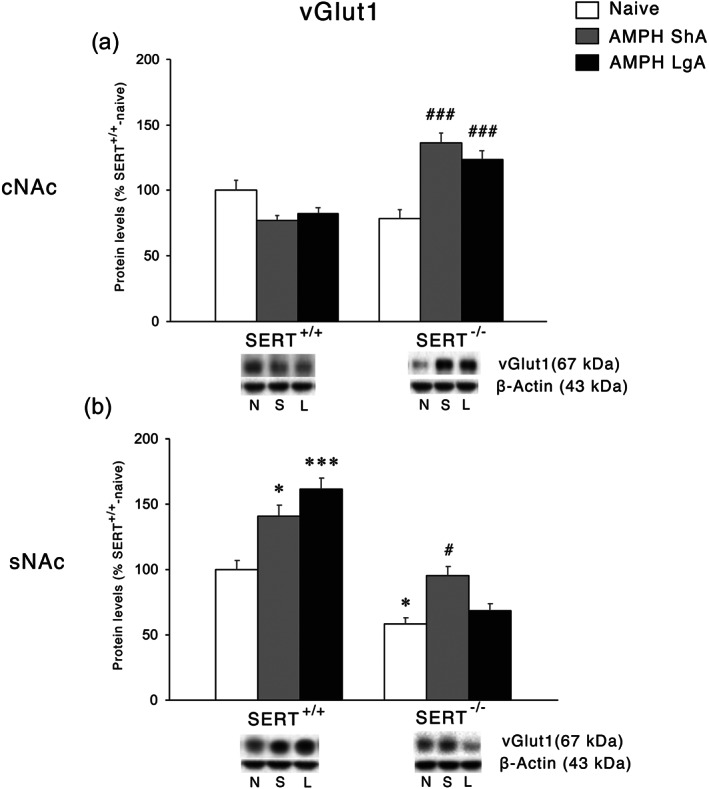
Interaction between SERT deletion and AMPH self‐administration on the vesicular glutamate transporter 1 (vGlut1) in the cNAc and sNAc. Protein levels of vGlut1 in (a) cNAc and (b) sNAc are expressed as percentages of SERT^+/+^‐naive rats. Below the graphs, representative immunoblots are shown for vGlut1 (60 kDa) and β‐Actin (43 kDa) proteins in the homogenate of cNAc and sNAc. Histograms represent the mean ± SEM of the following number of rats: naïve (SERT^+/+^ *n* = 6; SERT^−/−^ *n* = 6), ShA (SERT^+/+^ *n* = 14; SERT^−/−^ *n* = 13), and LgA (SERT^+/+^ *n* = 12; SERT^−/−^ *n* = 12). ^*^ *P* < 0.05, ^***^ *P* < 0.001 versus SERT^+/+^‐naive; ^#^ *P* < 0.05, ^###^ *P* < 0.001 versus SERT^−/−^‐naive (Tukey's multiple comparisons test). N, naïve; S, amphetamine short access; L, amphetamine long access

We first focused our attention on the presynaptic terminal by analysing the protein expression of the vGlut1 that is responsible of glutamate storage in presynaptic vesicles, and it is considered an indirect index of glutamate release from the presynaptic neuron to the extracellular space (El Mestikawy et al., 2011). In the cNAc, two‐way ANOVA revealed a main effect of AMPH access, genotype, and an AMPH access × genotype interaction (Figure 4a). Further intergroup subtesting revealed increased expression of vGlut1 in SERT^−/−^ rats exposed to either ShA or LgA with no effects in SERT^+/+^ rats following the same procedures.

In the sNAc, two‐way ANOVA revealed a main effect of AMPH access, genotype, and an AMPH access × genotype interaction (Figure 4b). In this subregion of the NAc, vGlut1 expression was reduced in SERT^−/−^‐naïve rats. The lack of SERT influenced the response to the different regimens of AMPH exposure. In fact, while vGlut1 protein levels in SERT^−/−^ were increased following ShA but not LgA to AMPH, in SERT^+/+^ rats, the expression of vGlut1 was increased independently from the ShA or LgA procedure.

### 3.4 Effect of ShA and LgA AMPH self‐administration on glial cells in SERT^+/+^ and SERT^−/−^ rats

**FIGURE 5:**
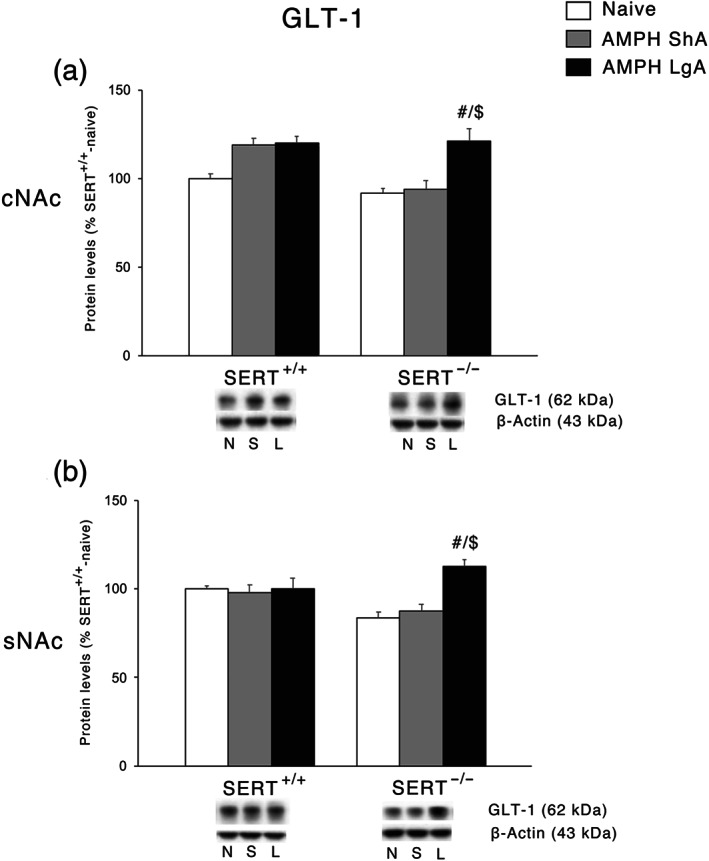
Interaction between SERT deletion and AMPH self‐administration on the glial glutamate transporter 1 (GLT‐1) in the cNAc and sNAc. Protein levels of GLT‐1 in (a) cNAc and (b) sNAc are expressed as percentages of SERT^+/+^‐naive rats. Below the graphs, representative immunoblots are shown for GLT‐1 (62 kDa) and β‐Actin (43 kDa) proteins in the homogenate of cNAc and sNAc. Histograms represent the mean ± SEM of the following number of rats: naïve (SERT^+/+^ *n* = 6; SERT^−/−^ *n* = 6), ShA (SERT^+/+^ *n* = 14; SERT^−/−^ *n* = 13), and LgA (SERT^+/+^ *n* = 12; SERT^−/−^ *n* = 12). ^#^ *P* < 0.05 versus SERT^−/−^‐naïve; ^$$^ *P* < 0.01 versus SERT^−/−^‐ShA (Tukey's multiple comparisons test). N, naïve; S, amphetamine short access; L, amphetamine long access

We next investigated the protein expression of the glial GLT‐1 that is responsible of the reuptake of glutamate back into the glial cells from the extracellular space (Roberts‐Wolfe & Kalivas, 2015). In the cNAc, two‐way ANOVA revealed a significant AMPH access effect and an effect of genotype whereas in the sNAc, an effect of AMPH access, but no genotype effect was found. In both cNAc and sNAc (Figures 5a and b, respectively), two‐way ANOVA revealed a significant AMPH access × genotype interaction. Examining the individual treatment effects, we found that SERT deletion in both cNAc and sNAc led to a significant increase of GLT‐1 expression in response to the LgA but not to the ShA procedure, whereas no significant effect was observed in SERT^+/+^rats.

### 3.5 Effect of ShA and LgA AMPH self‐administration on NMDA receptor subunits and the respective scaffolding proteins in the postsynaptic terminal of SERT^+/+^ and SERT^−/−^ rats

**FIGURE 6:**
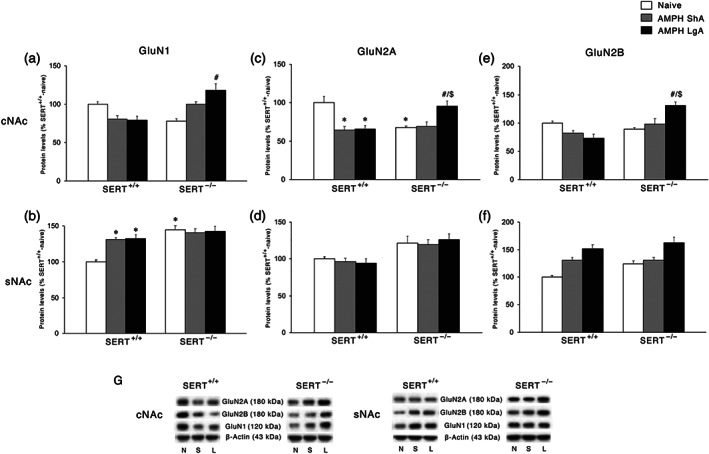
Interaction between SERT deletion and AMPH self‐administration on the NMDA receptor subunits in the cNAc and sNAc. Data show GluN1 levels in (a) cNAc and (b) sNAc, GluN2A levels in (c) cNAc and (d) sNAc, and GluN2B levels in (e) cNAc and (f) sNAc. In the lower panel (g), representative immunoblots are shown for GluN2A (180 kDa), GluN2B (180 kDa), GluN1 (120 kDa), and β‐Actin (43 kDa) proteins in the cNAc and sNAc homogenates of SERT^+/+^ and SERT^−/−^ rats exposed to AMPH. Results are expressed as percentages of SERT^+/+^‐naive rats. Histograms represent the mean ± SEM of the following number of rats: naïve (SERT^+/+^ *n* = 6; SERT^−/−^ *n* = 6), ShA (SERT^+/+^ *n* = 14; SERT^−/−^ *n* = 13), and LgA (SERT^+/+^ *n* = 12; SERT^−/−^ *n* = 12). ^*^ *P* < 0.05, ^**^ *P* < 0.01, ^***^ *P* < 0.001 versus SERT^+/+^‐naïve; ^#^ *P* < 0.05, ^##^ *P* < 0.01 versus SERT^−/−^‐naïve; ^$^ *P* < 0.05, ^$$^ *P* < 0.01 versus SERT^−/−^‐ShA (Tukey's multiple comparisons test). N, naïve; S, amphetamine short access; L, amph long access

The next step was to investigate the protein expression of the main subunits of the NMDA receptors. In the cNAc, two‐way ANOVA of GluN1 levels, the obligatory subunit of NMDA receptors, revealed an effect of genotype and a significant AMPH access × genotype interaction (Figure 6a). Post hoc testing revealed that the expression of this subunit of the NMDA receptor is significantly increased following the LgA but not the ShA procedure in SERT^−/−^ rats. Neither ShA, nor LgA, affected GluN1 expression in the cNAc of in SERT^+/+^ rats. A different situation was, instead, observed in the sNAc. Two‐way ANOVA revealed a main effect of AMPH access, genotype, and a significant AMPH access × genotype interaction (Figure 6b). Upon subtesting, we found an increased expression of GluN1 in SERT^−/−^‐naïve rats, which was not further enhanced by the ShA and the LgA procedure. Conversely, both the ShA and LgA conditions caused an enhancement in GluN1 protein levels in SERT^+/+^ rats.

We then investigated the expression of the accessory subunits of the NMDA receptor, that is, GluN2A and GluN2B. In the cNAc, two‐way ANOVA of GluN2A levels revealed a main effect of AMPH access and an AMPH access × genotype interaction effect (Figure 6c); in the same subregion, for the GluN2B subunit, two‐way ANOVA revealed a main effect of genotype and an AMPH access × genotype interaction (Figure 6e). GluN2A subunit expression was reduced in SERT^−/−^‐naïve rats; further, the post hoc intergroup comparisons revealed that the removal of SERT caused a different response to both the ShA and LgA conditions. In fact, GluN2A protein levels were reduced following both ShA and LgA AMPH self‐administration in SERT^+/+^ rats, whereas in SERT^−/−^ rats, no changes were found for ShA, while GluN2A protein levels were up‐regulated following LgA. GluN2B levels were not altered in AMPH‐exposed SERT^+/+^ rats. In SERT^−/−^ rats similarly to GluN2A, we found that the LgA, but not the ShA, procedure significantly up‐regulated the expression of this subunit.

In the sNAc, two‐way ANOVA revealed only a significant genotype effect (Figure 6d) for GluN2A and a significant AMPH access effect (Figure 6f) for GluN2B.

**FIGURE 7:**
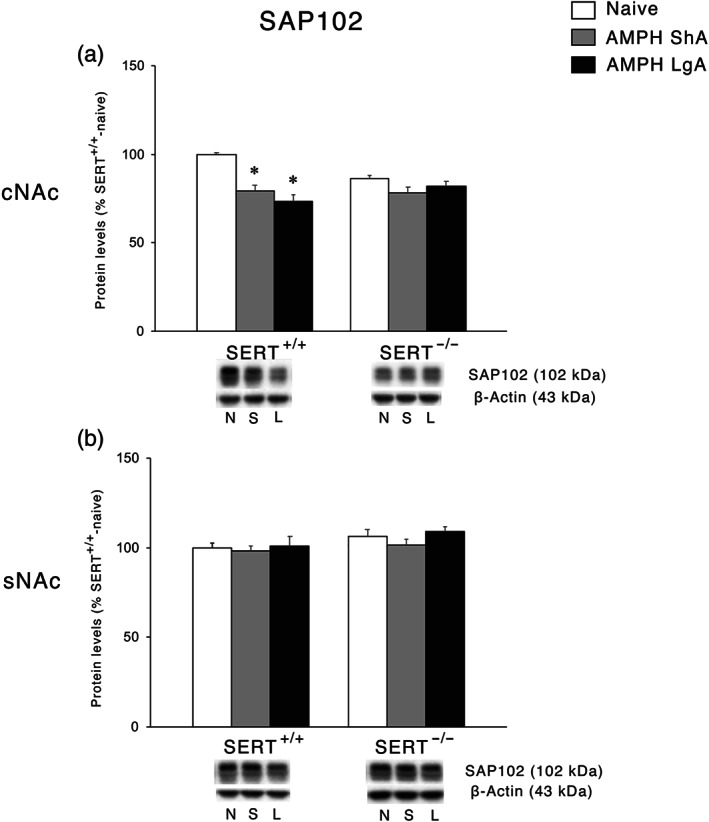
Interaction between SERT deletion and AMPH self‐administration on the scaffolding protein SAP102 in the cNAc and sNAc. Data show protein levels of SAP102 in the (a) cNAc and (b) sNAc expressed as percentage of SERT^+/+^‐naive rats. Below the graphs, representative immunoblots are shown for SAP102 (102 kDa) and β‐Actin (43 kDa) proteins in the homogenate of cNAc and sNAc. Histograms represent the mean ± SEM of the following number of rats: naïve (SERT^+/+^ *n* = 6; SERT^−/−^ *n* = 6), ShA (SERT^+/+^ *n* = 14; SERT^−/−^ *n* = 13), and LgA (SERT^+/+^ *n* = 12; SERT^−/−^ *n* = 12). ^**^ *P* < 0.01, ^***^ *P* < 0.001 versus SERT^+/+^‐naïve (Tukey's multiple comparisons test). N, naïve; S, amphetamine short access; L, amphetamine long access

Following the analysis of the NMDA receptor subunits, we investigated the expression of the main scaffolding protein of NMDA receptors, SAP102 (Won, Levy, Nicoll, & Roche, 2017). In the cNAc, two‐way ANOVA revealed a main effect of AMPH access and an AMPH access × genotype interaction (Figure 7a). Post hoc testing of the main treatment effects showed that both ShA and LgA AMPH self‐administration resulted in reduced SAP102 expression in SERT^+/+^ rats, whereas no changes were observed under both experimental conditions in SERT^−/−^ rats. In the sNAc, no significant changes in SAP102 levels were observed (Figure 7b).

### 3.6 Effect of ShA and LgA AMPH self‐administration on AMPA receptor subunits and their respective scaffolding proteins in the postsynaptic terminal of SERT^+/+^ and SERT^−/−^ rats

**FIGURE 8:**
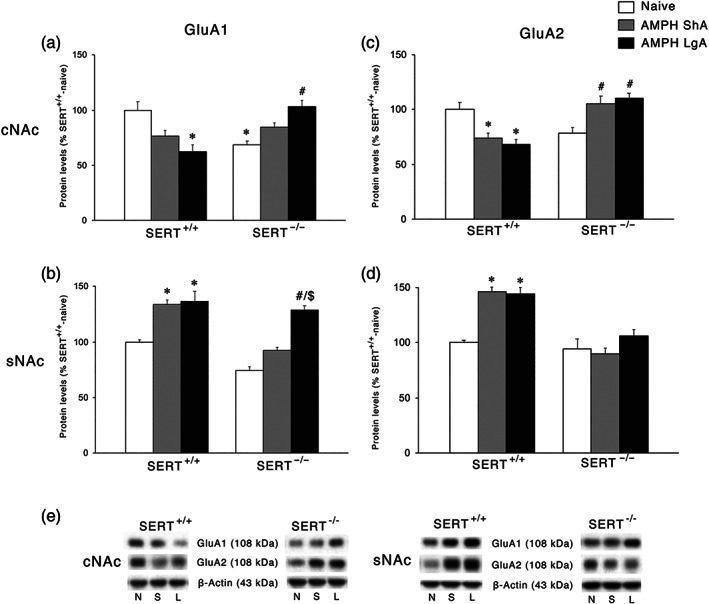
Interaction between SERT deletion and AMPH self‐administration on the AMPA receptor subunits in the cNAc and sNAc. Data show GluA1 levels in the (a) cNAc and (b) sNAc and GluA2 levels in the (c) cNAc and (d) sNAc. In the lower panel (e), representative immunoblots are shown for GluA1 (108 kDa), GluA2 (108 kDa), and β‐Actin (43 kDa) proteins in the cNAc and sNAc homogenates of SERT^+/+^ and SERT^−/−^ rats exposed to AMPH. Results are expressed as percentages of SERT^+/+^‐naive rats. Histograms represent the mean ± SEM of the following number of rats: naïve (SERT^+/+^ *n* = 6; SERT^−/−^ *n* = 6), ShA (SERT^+/+^ *n* = 14; SERT^−/−^ *n* = 13), and LgA (SERT^+/+^ *n* = 12; SERT^−/−^ *n* = 12). ^*^ *P* < 0.05, ^**^ *P* < 0.01 versus SERT^+/+^‐naïve; ^#^ *P* < 0.05, ^##^ *P* < 0.01, ^###^ *P* < 0.001 versus SERT^−/−^‐naïve; ^$$^ *P* < 0.01 versus SERT^−/−^‐ShA (Tukey's multiple comparisons test). N, naïve; S, amphetamine short access; L, amphetamine long access

We then moved to the analysis of the expression of the main subunits of the AMPA receptor, that is, GluA1 and GluA2. In the cNAc, for both GluA1 and GluA2 subunits (Figures 8a and b, respectively), two‐way ANOVA revealed a significant AMPH access × genotype interaction. For GluA2, two‐way ANOVA revealed also a main effect of genotype. SERT knockout reduced GluA1, but not GluA2, levels. The LgA condition reduced GluA1 levels in SERT^+/+^ while increasing it in SERT^−/−^. GluA2 levels were reduced following both ShA and LgA AMPH self‐administration in SERT^+/+^, while deletion of SERT increased GluA2 levels following both ShA and LgA AMPH self‐administration. In the sNAc, two‐way ANOVA of both GluA1 and GluA2 AMPA subunits revealed a main effect of AMPH access, genotype, and an AMPH access × genotype interaction effect (GluA1: Figure 8b; GluA2: Figure 8d). Both ShA and LgA conditions resulted in an elevation in GluA1 and GluA2 protein levels in SERT^+/+^ rats, whereas in SERT^−/−^ rats, no changes were observed with the exception of the increased expression of GluA1 following LgA.

**FIGURE 9:**
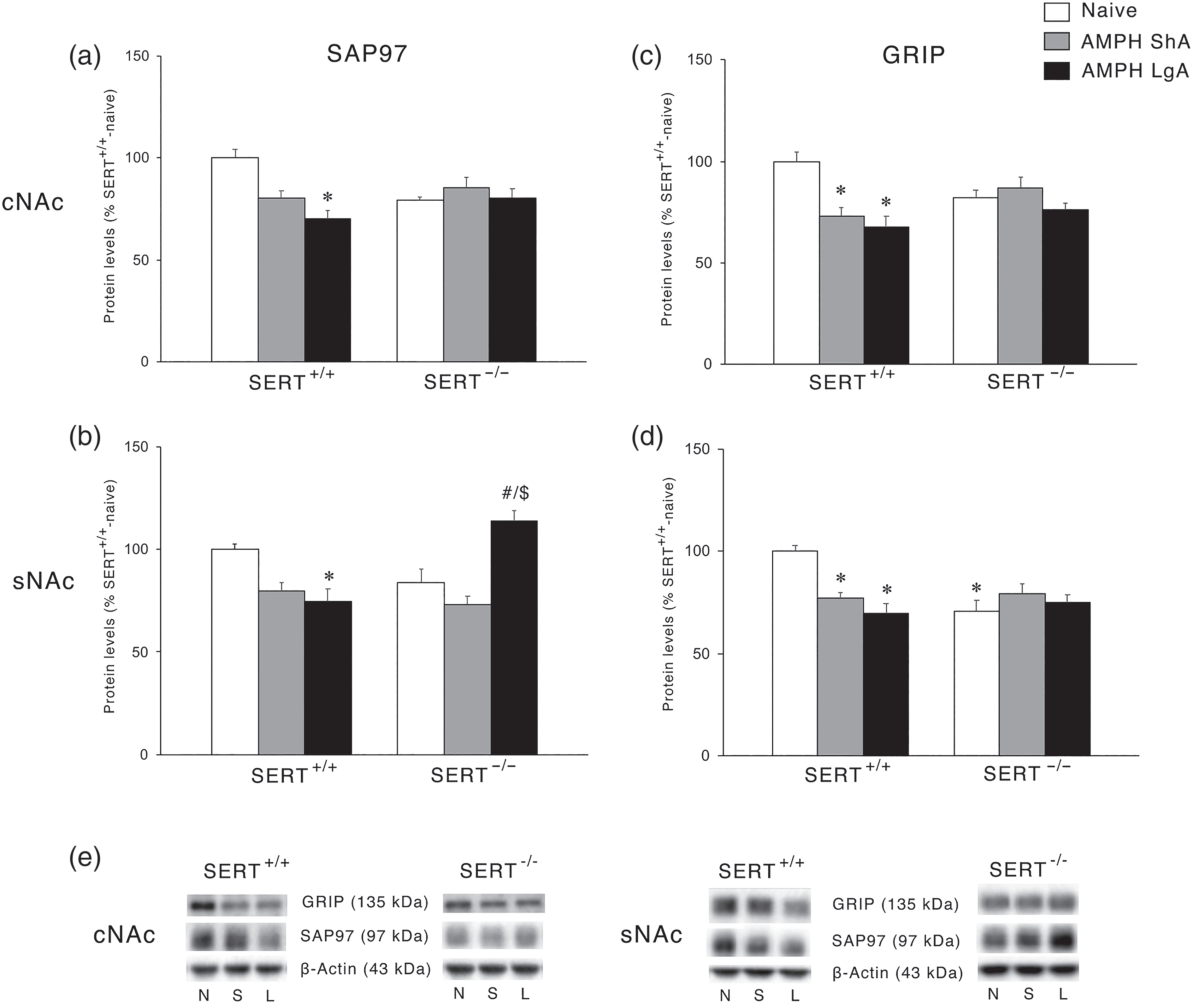
Interaction between SERT deletion and AMPH self‐administration on the scaffolding proteins SAP97 and GRIP in the cNAc and sNAc. Data show SAP97 levels in the (a) cNAc and (b) sNAc and GRIP levels in the (c) cNAc and (d) sNAc. In the lower panel (e), representative immunoblots are shown for GRIP (135 kDa), SAP97 (97 kDa), and β‐Actin (43 kDa) proteins in the cNAc and sNAc homogenates of SERT^+/+^ and SERT^−/−^ rats exposed to AMPH. Results are expressed as percentage of SERT^+/+^‐naive rats. Histograms represent the mean ± SEM of the following number of rats: naïve (SERT^+/+^ *n* = 6; SERT^−/−^ *n* = 6), ShA (SERT^+/+^ *n* = 14; SERT^−/−^ *n* = 13), and LgA (SERT^+/+^ *n* = 12; SERT^−/−^ *n* = 12). ^*^ *P* < 0.05, ^**^ *P* < 0.01, ^***^ *P* < 0.001 versus SERT^+/+^‐naïve; ^##^ *P* < 0.01 versus SERT^−/−^‐naïve; ^$$$^ *P* < 0.001 versus SERT^−/−^‐ShA (Tukey's multiple comparisons test). N, naïve; S, amphetamine short access; L, amphetamine long access

We then examined the expression of the scaffolding proteins specific for AMPA receptor subunits GluA1 and GluA2, that is, SAP97 and GRIP, respectively (Won et al., 2017). In the cNAc, for both SAP97 and GRIP (Figures 9a and c, respectively), two‐way ANOVA revealed a main effect of AMPH access and an AMPH access × genotype interaction. In SERT^+/+^ rats, we found a significant reduction of SAP97 under LgA AMPH self‐administration conditions only, whereas GRIP levels were reduced under both ShA and LgA conditions. Conversely, under both experimental conditions no significant changes were shown in SERT^−/−^ rats. In the sNAc, two‐way ANOVA of both SAP97 and GRIP levels revealed a main effect of AMPH access and an AMPH access × genotype interaction (SAP97: Figure 9b; GRIP: Figure 9d). Post hoc testing of the main treatment effects in SERT^+/+^ rats revealed reduced expression of SAP97 following LgA, but not ShA, procedures, whereas GRIP was similarly reduced by both experimental conditions. Conversely, in SERT^−/−^ rats, we found an increase in the expression of SAP97 following the LgA, but not the ShA, condition, whereas GRIP expression was decreased in SERT^−/−^‐naïve rats without further changes following both ShA and LgA AMPH self‐administration.

## 4 DISCUSSION

**FIGURE 10:**
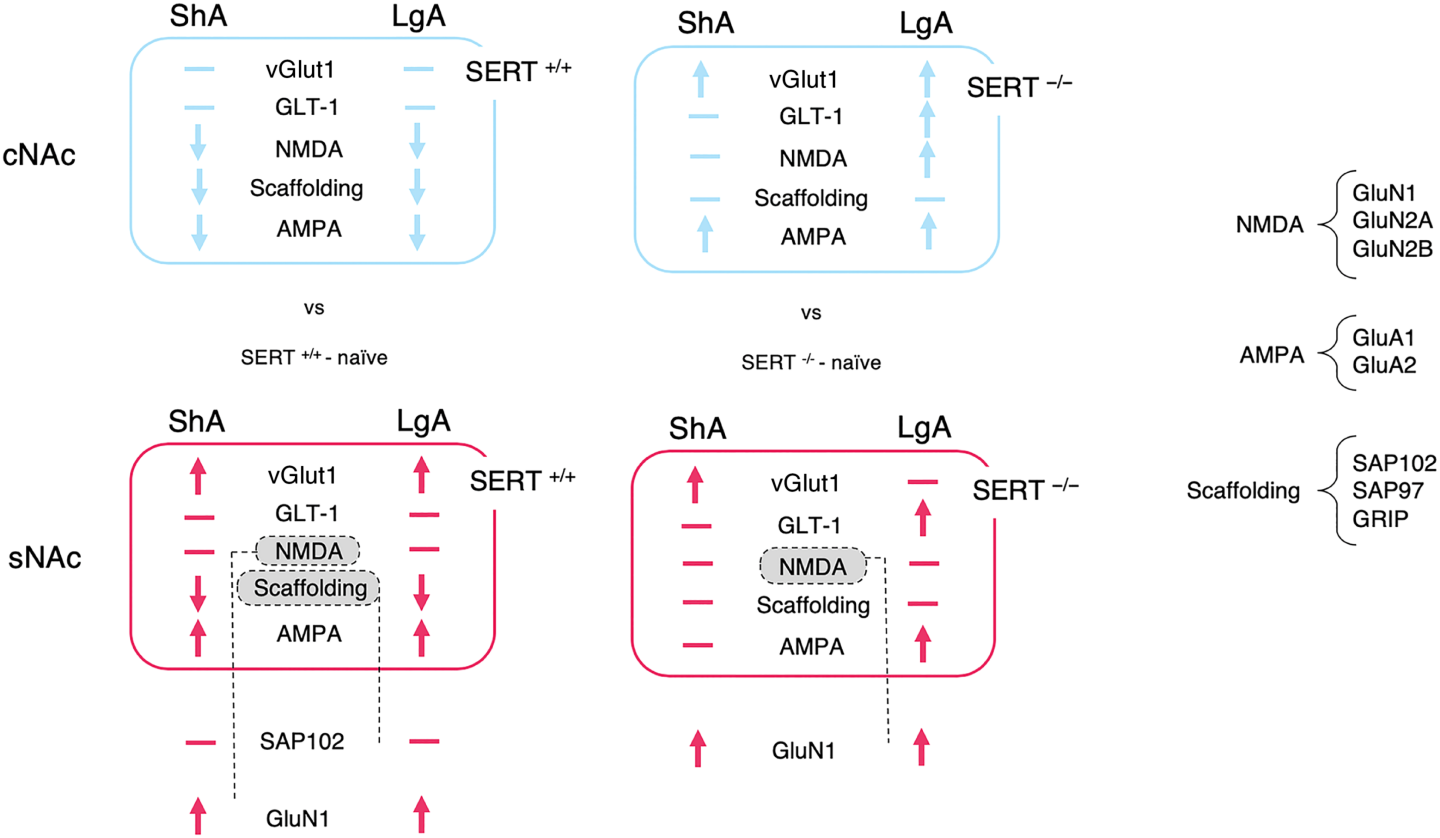
Summary of the protein expression findings in the nucleus accumbens shell and core of naïve SERT^+/+^ and SERT^−/−^ rats versus SERT^+/+^ and SERT^−/−^ rats exposed to ShA and LgA cocaine self‐administration. cNAC, nucleus accumbens core; sNAC, nucleus accumbens shell, ShA, amphetamine short access; LgA, amphetamine long access; vGlut1, vesicular glutamate transporter 1; GLT‐1, glial glutamate transporter 1; Scaffolding, scaffolding protein (e.g., SAP102)

We found that SERT^−/−^ rats displayed increased AMPH intake under LgA, but not ShA, conditions. Furthermore, the psychomotor response to AMPH was significantly higher in SERT^−/−^ rats. Finally, we found that after 24 h of withdrawal from AMPH self‐administration, the glutamatergic synapse was affected differently in SERT^−/−^ versus SERT^+/+^ rats, in a way that heavily relies on the subregion of the NAc (shell or core) and the type of AMPH access (ShA or LgA) (Figure 10).

### 4.1 AMPH's psychomotor and reinforcing effects

Because of the pronounced genotype differences in the psychomotor effects of AMPH, one could argue that locomotor activity had an influence on the observed genotype differences after LgA to AMPH self‐administration. However, the lack of genotype differences in AMPH intake under ShA condition and in the number of incorrect responses suggests that the increased AMPH intake under LgA condition in SERT^−/−^ versus SERT^+/+^ rats did not result from increased AMPH‐induced locomotor activity. Interestingly, while AMPH self‐administration was only increased in SERT^−/−^ rats under LgA but not ShA conditions, cocaine self‐administration was increased in these animals under both conditions (Verheij et al., 2018). While we did not conduct a direct comparison study, this implies that the serotonin influence on AMPH and cocaine self‐administration is different. A potential explanation is that AMPH has a lower affinity for SERT than cocaine (Howell & Kimmel, 2008) and that serotonin, next to dopamine, contributes to psychostimulant's reinforcing effects too. Given the lack of a genotype difference in the cocaine‐ (Verheij et al., 2014) and AMPH‐induced (unpublished data) dopamine release in the NAc, the observed differences in the psychomotor response to AMPH and AMPH self‐administration shown by SERT^+/+^ versus SERT^−/−^ rats are not likely related to the dopamine system.

### 4.2 AMPH effects in sNAc

Elevated serotonin levels can alter neurodevelopmental processes (Garcia et al., 2019; Homberg, Schubert, & Gaspar, 2010; Witteveen et al., 2013) and induce changes in neurotransmission (Brivio, Homberg, Riva, & Calabrese, 2019; Calabrese et al., 2013; Guidotti et al., 2012) through, for example, altered stimulation of serotonin receptors (e.g., Hall, Sora, Hen, & Uhl, ). Both routes can lead to alterations in the glutamate signalling in the NAc. Twenty‐four hours into withdrawal from AMPH self‐administration, we found that vGlut1 expression was reduced at baseline in the sNAc of SERT^−/−^ rats compared to SERT^+/+^ rats, suggesting reduced vesicular release of glutamate in SERT^−/−^ rats with consequently lower extracellular levels in the synapse. Of note, SERT^+/+^ rats showed increased vGlut1 expression after both ShA and LgA, whereas SERT^−/−^ rats exhibited an increase of the vesicular transporter following ShA and no change following LgA. Since the extracellular levels of glutamate are determined by a balance between its release and reuptake, the evidence that the accumbal expression of the plasmalemmal glutamate transporter GLT‐1 is not altered by AMPH in SERT^+/+^ rats might be indicative of an increased AMPH‐induced overflow of glutamate in the sNAc of these animals. Instead, GLT‐1 expression is increased in SERT^−/−^ LgA rats, possibly reducing the extracellular levels of glutamate, whereas it is not changed following ShA. This suggests that SERT deletion may be associated with an increased glutamate overflow as a consequence of a ShA‐induced increase of vGlut1. On the other hand, SERT deletion may reduce glutamate overflow as a consequence of a LgA‐induced increase of GLT‐1 expression. Since the sNAc is critical for the reinforcing properties of psychostimulants (Guillem et al., 2014), the possibility exists that altered extracellular glutamate levels in this brain subregion of SERT^−/−^ rats may affect drug seeking.

The analysis of the expression of glutamate receptors in the sNAc revealed differences between NMDA and AMPA receptors. At baseline, a significant change was represented by the increased expression of GluN1 in SERT^−/−^ rats, perhaps resulting from the reduced glutamate release caused by the decreased expression of vGlut1. Following both ShA and LgA AMPH self‐administration, we found increased expression of GluN1 in SERT^+/+^ rats, suggesting that the proposed higher release of glutamate in SERT^+/+^ rats is not compensated by a significant reduction in the main NMDA subunit. Conversely, in SERT^−/−^ rats neither ShA nor LgA is able to further enhance the expression of GluN1, suggesting that SERT deletion‐induced changes in GluN1 expression may be maximal. Notably, the observed increase in GluN1 expression were not accompanied by an increase in the levels of the accessory subunits GluN2A and GluN2B and of its anchoring protein SAP102, suggesting receptor instability following both ShA and LgA.

Regarding AMPA receptors, we found no significant changes at baseline between the two genotypes. GluA1 and GluA2 expression was up‐regulated following either ShA or LgA in SERT^+/+^ rats. However, when analysing the main scaffolding proteins for these two AMPA subunits, we found reduced expression of both SAP97 and GRIP in SERT^+/+^ rats. These findings suggest that GluA1 and GluA2 receptors lose their stability following both drug regimens in SERT^+/+^ rats. In SERT^−/−^ rats, both GluA1 and SAP97 expressions were significantly increased following LgA drug exposure, suggesting that LgA to AMPH increased the stability of at least the GluA1 receptor in these animals.

In SERT^+/+^ rats, the proposed AMPH‐induced increased release of glutamate, not compensated by changes in the expression of GLT‐1, leads to an increased expression of the main glutamate receptors. Instead, the localization and, presumably, the functionality of these receptors is attenuated by the uncoupling of their respective auxiliary proteins independently from the duration of the AMPH exposure. Conversely, in SERT^−/−^ rats, the proposed reduced glutamate release is accompanied by enhanced reuptake only following LgA drug exposure, a combination that leads to a functional compensation only for GluA1 but not for GluA2 AMPA subunit nor for the different NMDA receptor subunits. Of note, the increased expression of GluA1, but not GluA2, AMPA subunit following LgA may lead to the formation of GluA2‐lacking and Ca^2+^‐permeable AMPA receptors, a mechanism reported to drive addiction (Wolf, 2016).

### 4.3 AMPH effects in cNAc

A different situation was observed in the cNAc. In fact, neither expression of protein regulating release nor expression of protein regulating reuptake of glutamate was significantly changed at baseline in SERT^−/−^ rats compared to their wild‐type counterpart. In addition, no significant changes in these proteins were observed in the cNAc of SERT^+/+^ rats exposed to either regimen of AMPH. Conversely, both ShA and LgA may have promoted a significant higher release of glutamate that was accompanied by a higher uptake in the LgA, but not ShA, exposed in SERT^−/−^ rats. The up‐regulation of GLT‐1 may represent an adaptive mechanism to buffer the increased release of glutamate, which is absent in ShA‐exposed rats. This may indicate that changes in GLT‐1 expression reflect compensation in the core and synergism in the shell (see above). Interestingly, in the cNAc of SERT^+/+^ rats, we noted that both ShA and LgA to AMPH reduced the expression of GluN2 and GluA2 receptor subunits, an effect observed also for their respective scaffolding proteins, that is, SAP102, SAP97, and GRIP. Conversely, in SERT^−/−^ rats, LgA, but not ShA, significantly up‐regulated the three GluN and the two GluA subunits (with the exception of GluA2 in ShA‐exposed rats), an effect that was not accompanied, though, by a parallel enhancement of the expression of their respective scaffolding proteins, pointing towards an AMPH‐induced reduction in the stability of glutamatergic synapse of the cNAc of SERT^−/−^ rats.

## 5 CONCLUSION

Our data show that SERT removal and AMPH self‐administration converge on a common set of glutamate abnormalities that may contribute to shape both the behavioural and molecular effects herein observed. Changes in glutamate signalling fit the glutamate hypothesis for drug addiction (Kalivas, 2009). The data show that the combination of SERT deletion and AMPH exposure leads to effects that are unique and distinguishable from those of the individual experimental conditions. Accordingly, we can make several considerations: (a) the glutamate system is differently regulated by AMPH in SERT^−/−^ rats when compared to SERT^+/+^ rats; (b) the molecular determinants of the glutamate synapse are differently regulated in cNAc and sNAc, confirming that these two subregions have their own function in regulating drug dependence; and (c) the LgA paradigm shapes the glutamatergic synapse differently from the ShA condition. Notably, because the NAc is tightly connected to various other brain regions, including the PFC, thalamus, amygdala, and hippocampus also playing important roles in drug addiction (Noori, Spanagel, & Hansson, 2012), it is very likely that, beyond the NAc, molecular changes in connected areas are also implicated in the observed individual differences in responsivity to AMPH. Because we only tested male rats, further research is required to determine whether these findings extend to female rats. Taken together, the perturbation of serotonin activity may, via dysregulation of glutamate homeostasis in these NAc regions, contribute to drive the transition from goal‐directed drug intake towards addictive states. Hence, we propose that manipulation of serotonergic and/or glutamatergic systems may affect not only cocaine but also AMPH dependence.

## AUTHOR CONTRIBUTIONS

L.C. conducted the western blot experiments, analysed the data, and contributed to the writing of the manuscript. M.V. conducted the self‐administration experiment, made figures for the self‐administration data, and contributed to the writing of the manuscript. K.R. initiated the writing of the manuscript and generated figures. G.T. contributed to sample preparation and conducted the western blot experiments. F.M. conducted the western blot analyses and made the graphic abstract and overview figure. P.P. supervised the psychomotor experiment and the project. A.N. supervised the psychomotor experiment and contributed to the writing of the manuscript. J.G. conducted the psychomotor experiment. F.F. conceived and planned the experiments, supervised the molecular analyses, contributed to the interpretation of the results, and contributed to the writing of the manuscript. J.R. conceived and planned the experiments, contributed to the writing of the manuscript, and supervised the project. All authors discussed the results and contributed to the final manuscript.

## CONFLICT OF INTEREST

The authors declare no conflicts of interest.

## DECLARATION OF TRANSPARENCY AND SCIENTIFIC RIGOUR

This Declaration acknowledges that this paper adheres to the principles for transparent reporting and scientific rigour of preclinical research as stated in the *BJP* guidelines for Natural Product Research, Design and Analysis, Immunoblotting and Immunochemistry, and Animal Experimentation, and as recommended by funding agencies, publishers and other organizations engaged with supporting research.

## Supporting information


**Figure S1** Number of incorrect lever presses (A, B) and timeout responses (C, D) during AMPH self‐administration in SERT^−/−^ and SERT^+/+^ rats under ShA and LgA conditions. No genotype differences were observed. Data are represented as mean ± SEM. n.s.: not significant.

**Table S1** F and p values of both behavioral and molecular data presented in the results section.
