# Supplementary figures and images for: Hypersensitivity to amphetamine's psychomotor and reinforcing effects in serotonin transporter knockout rats: Glutamate in the nucleus accumbens
Source: Br J Pharmacol. 2020 Aug 30;177(19):4532–47. doi: 10.1111/bph.15211 (PMC7484509; doi:10.1111/bph.15211)

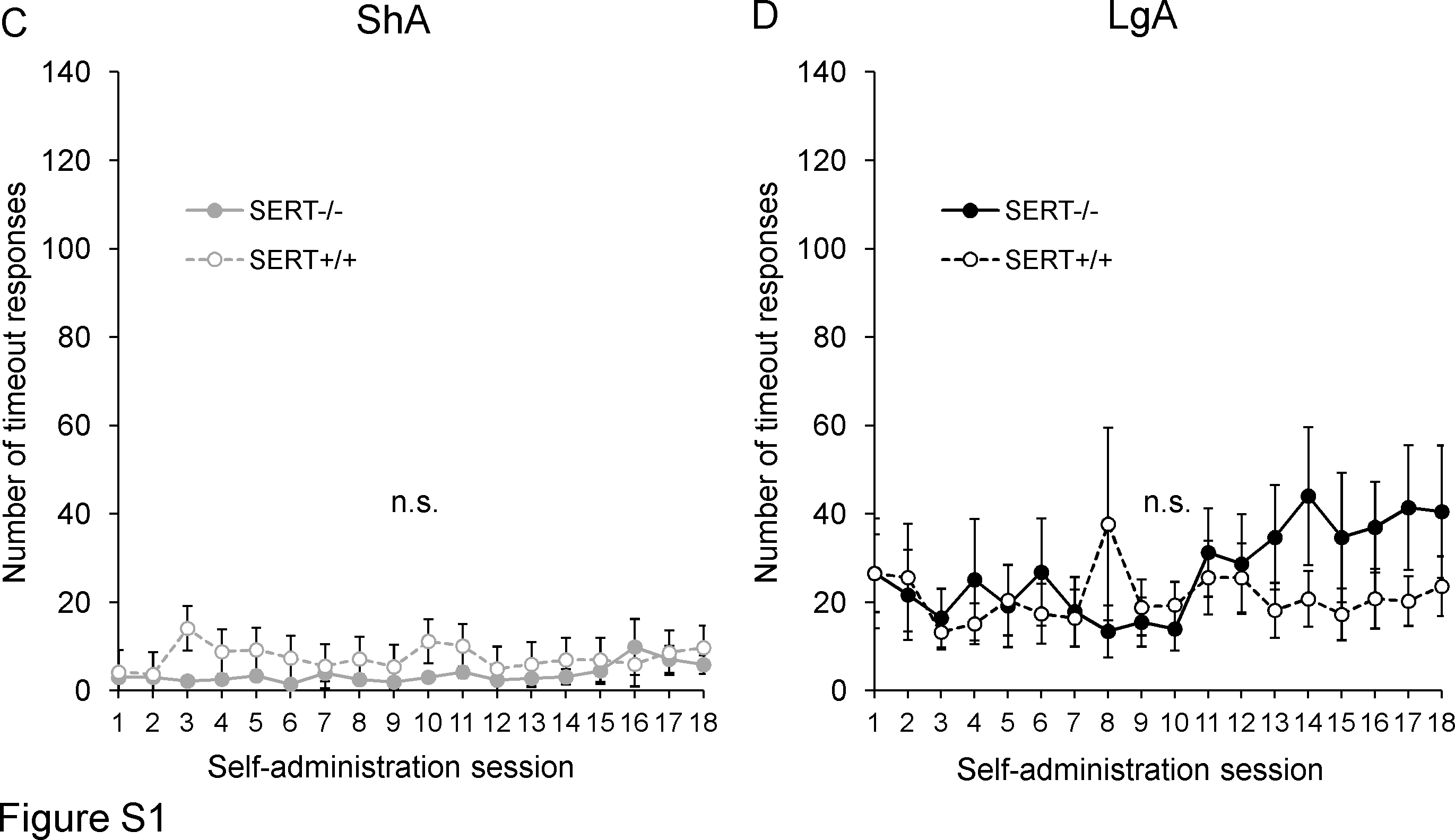

Supplement: Supplementary file 1 — Figure S1 Number of incorrect lever presses (A, B) and timeout responses (C, D) during AMPH self‐administration in SERT−/− and SERT+/+ rats under ShA and LgA conditions. No genotype differences were observed. Data are represented as mean ± SEM. n.s.: not significant. [file BPH-177-4532-s001.tif]
