# Supplementary material for: Hypersensitivity to amphetamine's psychomotor and reinforcing effects in serotonin transporter knockout rats: Glutamate in the nucleus accumbens
Source: Br J Pharmacol. 2020 Aug 30;177(19):4532–47. doi: 10.1111/bph.15211 (PMC7484509; doi:10.1111/bph.15211)
Supplement: Supplementary file 2 — Table S1 F and p values of both behavioral and molecular data presented in the results section. [file BPH-177-4532-s002.pdf]

| Figure(s) | Effect | F (df, error) | P |
|---|---|---|---|
| 2 | genotype x AMPH dose x time effect<br>(GG corrected) | $F_{(3.72,\ 169.63)} = 3.668$ | P < 0.01 |
| 2 | AMPH dose x time effect<br>(GG corrected) | $F_{(3.72,\ 169.63)} = 36.678$ | P < 0.0001 |
| 3A and 3B | genotype x access x self-administration session effect<br>(GG corrected) | $F_{(3.500,\ 175.012)} = 3.951$ | P < 0.01 |
| 3A vs 3B | access x self-administration session effect<br>(GG corrected) | $F_{(3.500,\ 175.012)} = 15.663$ | P < 0.001 |
| 3A | genotype x self-administration session effect<br>(GG corrected) | not significant: n.s. | P > 0.05 |
| 3B | genotype x self-administration session effect<br>(GG corrected) | $F_{(3.322,\ 83.058)} = 4.621$ | P < 0.01 |
| 4A | access,<br>genotype<br>access x genotype | $F_{(2,57)}=3.46$, p=0.038<br>$F_{(1,57)}=25.57$<br>$F_{(2,57)}=18.20$ | P < 0.05<br>P < 0.0001<br>P < 0.0001 |
| 4B | access,<br>genotype<br>access x genotype | $F_{(2,55)}=311.50$<br>$F_{(1,55)}=83.97$<br>$F_{(2,55)}=7.425$ | P < 0.0001<br>P < 0.0001<br>P < 0.01 |
| 5A | access,<br>genotype<br>access x genotype | $F_{(2,57)}=10.94$<br>$F_{(1,57)}=9.349$<br>$F_{(2,57)}=3.444$ | P < 0.0001<br>P < 0.01<br>P < 0.05 |
| 5B | access,<br>access x genotype | $F_{(2,56)}=5.055$<br>$F_{(2,56)}=3.347$ | P < 0.01<br>P < 0.05 |
| 6A | genotype,<br>access x genotype | $F_{(1,57)}=4.544$<br>$F_{(2,57)}=8.103$ | P < 0.05<br>P < 0.001 |
| 6B | access,<br>genotype<br>access x genotype | $F_{(2,53)}=3.314$<br>$F_{(1,53)}=20.53$<br>$F_{(2,53)}=4.675$ | P < 0.05<br>P < 0.0001<br>P < 0.05 |
| 6C | access,<br>access x genotype | $F_{(2,57)}=5.137$<br>$F_{(2,57)}=11.36$ | P < 0.01<br>P < 0.0001 |
| 6D | genotype | $F_{(1,55)}=19.76$ | P < 0.0001 |
| 6E | genotype,<br>access x genotype | $F_{(1,56)}=12.21$<br>$F_{(2,56)}=10.41$ | P < 0.001<br>P < 0.001 |
| 6F | access | $F_{(2,56)}=15.03$ | P < 0.0001 |
| 7A | access,<br>access x genotype | $F_{(2,56)}=9.318$<br>$F_{(2,56)}=4.476$ | P < 0.001<br>P < 0.05 |
| 8A | access x genotype | $F_{(2,57)}=16.03$ | P < 0.0001 |
| 8B | access,<br>genotype<br>access x genotype | $F_{(2,57)}=17.17$<br>$F_{(1,57)}=17.81$<br>$F_{(2,57)}=3.571$ | P < 0.0001<br>P < 0.0001<br>P < 0.05 |
| 8C | genotype,<br>access x genotype | $F_{(1,57)}=13.09$<br>$F_{(2,57)}=13.79$ | P < 0.001<br>P < 0.0001 |
| 8D | access,<br>genotype<br>access x genotype | $F_{(2,58)}=4.995$<br>$F_{(1,58)}=15.09$<br>$F_{(2,58)}=3.301$ | P < 0.05<br>P < 0.001<br>P < 0.05 |
| 9A | access,<br>access x genotype | $F_{(2,57)}=4.464$<br>$F_{(2,57)}=5.152$ | P < 0.05<br>P < 0.01 |
| 9B | access,<br>access x genotype | $F_{(2,57)}=7.647$<br>$F_{(2,57)}=15.86$ | P < 0.01<br>P < 0.0001 |
| 9C | access,<br>access x genotype | $F_{(1,58)}=5.822$<br>$F_{(2,58)}=4.385$ | P < 0.01<br>P < 0.05 |
| 9D | access,<br>access x genotype | $F_{(1,57)}=3.795$<br>$F_{(2,57)}=7.230$ | P < 0.05<br>P < 0.01 |

**Supplementary table 1.** F and p values of both behavioral and molecular data presented in the results section.
